# Pediatric Bone Sarcomas: A Single-Center Experience in Jordan

**DOI:** 10.7759/cureus.109531

**Published:** 2026-05-24

**Authors:** Mais Jazazi, Mousa A Qatawneh, Ayman Alhwayan, Hanadi Alkhalaileh, Shatha Alqaisi, Firas Alsmadi, Eman Alquraan, Mohammad Alhawamdeh, Dima Alhamad, Maher Khader

**Affiliations:** 1 Pediatric Hematology-Oncology, Royal Medical Services, Queen Rania Al Abdullah Hospital for Children, Amman, JOR; 2 Pathology and Laboratory Medicine, Royal Medical Services, Princess Iman Center for Research and Laboratory Sciences, Amman, JOR; 3 Orthopedic Surgery, Royal Medical Services, Royal Rehabilitation Center, Amman, JOR; 4 Pediatrics/Neonatology, Royal Medical Services, Queen Rania Al Abdullah Hospital for Children, Amman, JOR

**Keywords:** bone tumors, ewing sarcoma, jordan, osteosarcoma, pediatric oncology

## Abstract

Background: Osteosarcoma and Ewing sarcoma are the most common primary malignant bone tumors in children and adolescents. This study aimed to characterize the demographic, clinicopathologic, treatment, and survival features of pediatric patients with these malignancies at a tertiary referral center in Jordan.

Methods: This retrospective cohort study included all patients aged 18 years or younger with histologically confirmed osteosarcoma or Ewing sarcoma treated at Queen Rania Al Abdullah Hospital for Children, Amman, Jordan, between January 2015 and December 2025. Data were extracted regarding patient demographics, clinical presentation, laboratory biomarkers, treatment protocols, and clinical outcomes. Descriptive statistics and binary logistic regression were utilized to identify predictors of relapse and mortality.

Results: The study cohort comprised 52 patients (mean age 10.5 years) of whom 30 (57.7%) were male. Ewing sarcoma accounted for 75% of cases (n=39), and osteosarcoma for 25% (n=13). During the study period, nine (17.3%) patients experienced relapse, and 13 (25%) succumbed to the disease. Multivariable logistic regression analysis was performed to identify predictors of relapse and mortality. No statistically significant predictors of relapse were identified in this cohort. Specifically, metastatic disease at diagnosis (OR 9.67; 95%CI, 2.21-42.33; p = 0.003) and positive surgical margins (OR 0.089 for negative vs positive margins; 95%CI, 0.015-0.538; p = 0.008) were both significantly associated with increased mortality.

Conclusions: In this single-center cohort, a high frequency of metastatic disease at presentation and positive surgical margins were strongly associated with increased mortality. These findings underscore the critical need for earlier clinical detection and for implementing robust local control strategies. Improving these elements of the care pathway is essential to enhancing survival outcomes in pediatric orthopedic oncology within the region.

## Introduction

Malignant bone tumors are uncommon in children, being just 3-6% of all childhood cancers [1]. Each year, there are about 9-10 new cases per million people aged under 20 years. Most of these tumors are either osteosarcoma (55-60%) or Ewing sarcoma (30-35%) [2]. Although these cancers are uncommon, bone and joint tumors are the third most common cause of pediatric cancer‑related death in some high‑income countries [3].

Clinical outcomes have improved significantly with multimodal therapy. The current standard of care includes multi-agent chemotherapy, with doxorubicin, cisplatin, and high-dose methotrexate commonly used for osteosarcoma, and vincristine, doxorubicin, cyclophosphamide, ifosfamide, and etoposide commonly used for Ewing sarcoma, along with surgery and radiotherapy when indicated [4,5]. Recent studies show that five-year overall survival is about 65-75% for osteosarcoma and 70-80% for Ewing sarcoma in patients with localized disease. However, children with metastatic disease have much lower five-year survival rates, usually around 20-30%. Even with better imaging, surgery, and treatments, overall survival has not improved much in recent years, especially for high-risk patients with metastatic, relapsed, or treatment-resistant disease [5,6].

Pediatric bone sarcoma patterns vary across regions and health systems, with delays in diagnosis and limited access to specialized care contributing to advanced presentation in some settings. Reports from Middle Eastern centers suggest that many children still present with advanced disease [7-9]. Therefore, this study aimed to characterize the demographic, clinicopathological, treatment, and survival features of pediatric patients with osteosarcoma and Ewing sarcoma managed at a tertiary referral center in Jordan.

## Materials and methods

This was a retrospective cohort study conducted at the Queen Rania Al Abdullah Hospital for Children, Amman, Jordan, the national referral center for pediatric oncology. Records of pediatric patients aged 18 years or younger diagnosed with osteosarcoma or Ewing sarcoma between January 2015 and December 2025 were reviewed. Follow-up was completed for all included patients by December 2025, which served as the censoring date. Overall survival and event-free survival were calculated from the date of diagnosis to death, first event, or last follow-up, as applicable. The study was approved by the Pharmacological, Clinical and Professional Ethics, Research and Studies Committee, General Command of the Jordanian Armed Forces of the Royal Medical Services (15-4/2026)

Study population

The study cohort comprised all pediatric patients (aged ≤18 years) with histologically confirmed primary osteosarcoma or Ewing sarcoma who received complete primary treatment, clinical follow-up, and surveillance imaging at Queen Rania Al Abdullah Hospital for Children. Patients were excluded if they refused the institutional chemotherapy protocol, transferred care to another facility, had primary extraosseous soft-tissue Ewing sarcoma, had secondary bone tumors, or had incomplete medical, diagnostic, or longitudinal follow-up records that prevented reliable outcome analysis. Queen Rania Al Abdullah Hospital for Children, the sole center treating military-insured pediatric cancer patients nationwide, serves as Jordan’s primary public-sector facility for osteosarcoma and Ewing sarcoma, minimizing selection bias in this cohort.

Data collection

Clinical data were retrospectively extracted from institutional electronic medical records. Patient variables included age at diagnosis, sex, histological subtype, primary tumor site, tumor size, clinical stage at diagnosis, metastatic sites, and presence of pathological fractures. Clinical stage was classified as localized or metastatic based on imaging findings from chest CT, MRI, and/or bone scintigraphy or PET-CT. Serum alkaline phosphatase (ALP) and neutrophil-to-lymphocyte ratio (NLR) were measured at diagnosis before treatment initiation and were analyzed as continuous variables.

Treatment-related variables included surgical approach (limb-salvage or amputation), surgical margin status, histologic response to neoadjuvant chemotherapy according to the Huvos classification, and receipt of radiotherapy. A good histologic response was defined as 90% or more tumor necrosis, whereas a poor response was defined as less than 90% necrosis. Disease relapse was defined by clinical or radiological evidence of new or progressive disease. Histopathologic confirmation was obtained in equivocal cases when feasible, but it was not mandatory when imaging and clinical findings were conclusive.

Outcome definitions

Overall survival (OS) was defined as the time from histopathologic diagnosis to death from any cause or the last known follow-up date for patients alive at the end of the study. Event-free survival (EFS) was defined as the time from diagnosis to the first occurrence of disease progression, local or distant relapse, or death from any cause. Patients without an event were censored at their last contact date.

Statistical analysis

Continuous variables were summarized as means ± standard deviations (SD) and ranges, while categorical variables were summarized as frequencies and percentages. Univariate descriptive analyses were performed to characterize the cohort with respect to demographics, clinicopathological features, treatment patterns, and outcomes. Multivariable binary logistic regression models were used to identify predictors of relapse and mortality based on final outcome status at last follow-up; Cox proportional hazards regression was not performed because the analysis did not include formal time-to-event modeling. Covariates included in the models were age at diagnosis, sex, histological subtype, tumor size, metastatic status at diagnosis, surgical margin status, receipt of radiotherapy, serum ALP, and NLR. Results are presented as OR with 95% CI and corresponding p-values. All statistical tests were two-sided, with significance set at p < 0.05. Data were analyzed using IBM SPSS Statistics for Windows, version 28.0 (IBM Corp., Armonk, New York, United States).

## Results

Patient and tumor characteristics

The study cohort comprised 52 pediatric patients. The mean age at diagnosis was 10.5 years, and the median age was 11 years (SD 3.84; range: 1-17 years), with a male predominance (n=30, 57.7%). Ewing sarcoma was the most frequent histological subtype (n=39, 75.0%), while osteosarcoma accounted for 25.0% (n=13). The most common primary tumor site was the pelvis (n=11, 21.2%), followed by the proximal tibia (n=9, 17.3%). The mean tumor size was 7.4 cm (SD 2.79; range: 1.5-17.0 cm).

At diagnosis, 32 (61.6%) patients presented with localized disease, while 20 (38.4%) presented with metastatic disease. Among patients with metastatic disease, pulmonary involvement was observed in 15/20 (75%) cases. Pathological fractures were present in seven (13.5%) of the cohort at diagnosis. Baseline laboratory markers showed wide variability: mean ALP was 389.4 U/L (range: 3.6-4435 U/L), and the mean NLR was 2.58 (range: 0.1-13.3). Baseline characteristics are summarized in Table 1.

**Table 1:** Baseline demographic, tumor, and laboratory characteristics (N=52) Data are given as frequency (percentage) except in age, tumor size, ALP, and NLR, where they are given as mean±SD (range) ALP: alkaline phosphatase; NLR: neutrophil-to-lymphocyte ratio *one patient's ALP at diagnosis was 4435 U/L

Variables	Category	Frequency (Percentage)
Age (years), mean±SD (range)		10.5±3.84 (1-17)
Gender	Male	30 (57.7)
	Female	22 (42.3)
Type	Osteosarcoma	13 (25.0)
	Ewing sarcoma	39 (75.0)
Primary site	Pelvis	11 (21.2)
	Distal femur	8 (15.4)
	Humerus	7 (13.5)
	Proximal tibia	9 (17.3)
	Radius	4 (7.7)
	Vertebral bodies	2 (3.8)
	Chest wall	4 (7.7)
	Skull	4 (7.7)
	Fibula	2 (3.8)
	Lung tissue	1 (1.9)
Tumor size (cm), mean±SD (range)		7.40±2.79 (1.5-17.0)
Tumor stage	Localized	32 (61.6)
	Metastatic	20 (38.4)
Site of metastasis n=20	Lung	15 (75)
	Other bones	4 (20)
	Bone marrow	1 (5)
Pathological fracture	No	45 (86.5)
	Yes	7 (13.5)
Alkaline phosphatase (U/L)		389.39±751.0 (3.6-4435*)
Neutrophil-to-lymphocyte ratio		2.58 ±2.76 (0.1-13.3)

Treatment characteristics

For local control, 20 (38.4%) of the 52 did not undergo surgery. Of the remaining 32, 29 (90.6%) patients underwent limb-salvage surgery, and three (9.4%) underwent amputation. Among the 32 patients with available surgical margin data, 17 (53.1%) had negative margins, and 15 (46.9%) had positive margins. Histologic response to neoadjuvant chemotherapy was assessed in operated patients, and 20/32 (62.5%) showed a good response, defined as 90% or more tumor necrosis, while 12/32 (37.5%) had a poor response, defined as less than 90% necrosis. Radiotherapy was administered to 25 (48.1%) patients, whereas 27 (51.9%) did not receive radiotherapy. Table 2 summarizes these treatment details.

**Table 2:** Local treatment modalities and radiotherapy treatment

Management	Category	Frequency (Percentage)
Surgical approach (n=32)	Limb salvage	29 (90.6)
	Amputation	3 (9.4)
Surgical margins (n=32)	Positive	15 (46.9)
	Negative	17 (53.1)
Tumor chemo necrosis percentage (n=32)	≥90%	20 (62.5)
	<90%	12 (37.5)
Radiation therapy	No	27 (51.9)
	Yes	25 (48.1)

Patient outcomes

During follow-up, 43 (82.7%) patients did not experience relapse. Among the nine patients with relapse, five (55.5%) had pulmonary relapse, three (33.3%) had local relapse, and one (11.2%) had liver relapse. The mean time to relapse was 16.56 ± 9.01 months (range, 5-37 months), and the median time to relapse was 13 months. At the last follow-up, 39 (75.0%) patients were alive, and 13 (25.0%) had died. For event-free survival, 27 patients were categorized as event-free or censored without an event, while 10 (37.1%) experienced progression, four (14.8%) had relapse, and 13 (48.1%) died. Table 3 summarizes these outcomes.

**Table 3:** Relapse , overall survival, and event free survival characteristics Data are given as frequency (percentage) except in time for relapse and overall survival, which are given as mean±SD (range)

Variables	Category	Frequency (Percentage)
Relapse	No	43 (82.7)
	Yes	9 (17.3)
Site of relapse (n=9)	Local	3 (33.3)
	Lung	5 (55.5)
	Liver	1 (11.2)
Time for relapse (months), mean±SD (range)		16.56±9.01 (5-37)
Mortality	Alive	39 (75.0)
	Dead	13 (25.0)
Overall survival (months), mean±SD (range)		14.69±10.04 (2-28)
Event-free survival (n= 27)	Progression	10 (37.1)
	Relapse	4 (14.8)
	Death	13 (48.1)

Predictors of relapse

In the multivariable logistic regression model assessing potential predictors of relapse, none of the examined variables reached statistical significance. Age at diagnosis, sex, histological subtype (Ewing vs osteosarcoma), tumor size, metastatic status, surgical margin status, receipt of radiotherapy, baseline ALP, and NLR all demonstrated ORs with 95% CIs that did not cross unity (p > 0.05). For example, tumor size (OR 1.17; 95% CI 0.96-1.43) and surgical margin status (OR 4.55; 95% CI 0.77-26.84 for negative vs positive margins) exhibited ORs greater than 1.0 but with wide 95% CIs that included unity, likely reflecting the limited sample size and the small number of relapse events. Detailed results are presented in Table 4.

**Table 4:** Multivariable logistic regression analysis of predictors of relapse in pediatric bone sarcoma B: slope of regression, SE: standard error, Sig: significance ( P-value). Reference categories: male sex, osteosarcoma, localized disease, positive margins, no radiotherapy.

Predictors for relapse	B	S.E.	Sig.	OR	95% C.I. for OR	
					Lower	Upper
Age at diagnosis (years)	0.055	0.105	0.598	1.057	0.861	1.297
Sex (females vs males)	0.648	0.740	0.381	1.912	0.448	8.150
Subtype (Ewing vs osteo)	0.185	0.875	0.833	1.203	0.217	6.681
Tumor size	0.158	0.100	0.115	1.171	0.963	1.425
Metastatic vs localized	-0.268	0.773	0.729	0.765	0.168	3.478
Surgical margins (negative vs positive)	1.515	0.905	0.094	4.550	0.771	26.835
Radiation (yes vs no)	-0.177	0.737	0.811	0.838	0.198	3.553
Alkaline phosphatase (U/L)	-0.002	0.003	0.539	0.998	0.993	1.003
Neutrophil-to-lymphocyte ratio	0.144	0.111	0.196	1.155	0.928	1.437

Predictors of mortality

In the multivariable logistic regression analysis, significant prognostic factors for mortality were identified. The presence of metastatic disease at diagnosis was strongly associated with increased mortality (OR 9.67; 95% CI 2.21-42.33; p = 0.003), indicating a nearly ten-fold increase in the odds of death compared to patients with localized disease; this is consistent with the well-established adverse prognostic impact of metastases in pediatric bone sarcomas. Furthermore, surgical margin status was significantly associated with mortality: achieving negative margins was associated with markedly reduced odds of death (OR 0.089; 95% CI 0.015-0.538; p = 0.008) compared to positive margins, underscoring the necessity of complete surgical resection. Other evaluated variables, including age, sex, tumor type, tumor size, receipt of radiotherapy, serum ALP, and NLR, did not reach statistical significance in this cohort. These findings are summarized in Table 5.

**Table 5:** Multivariable logistic regression analysis of predictors of mortality in pediatric bone sarcoma B: slope of regression, SE: standard error, Sig: significance ( P-value). Reference categories: male sex, osteosarcoma, localized disease, positive margins, no radiotherapy.

Predictors for death	B	S.E.	Sig.	OR	95% C.I. for OR	
					Lower	Upper
Age at Diagnosis (years)	-.038	0.082	0.644	0.963	0.820	1.131
Sex (females vs males)	-1.153	0.732	0.115	0.316	0.075	1.326
Type (Ewing vs osteo)	-0.393	0.711	0.580	0.675	0.168	2.720
Tumor size	0.003	0.086	0.974	1.003	0.847	1.187
Metastatic vs localized	2.269	0.754	0.003	9.667	2.207	42.334
Surgical margins (negative vs positive)	-2.420	0.919	0.008	0.089	0.015	0.538
Radiation (yes vs no)	-.521	0.654	0.425	0.594	0.165	2.139
Alkaline phosphatase (U/L)	0.000	0.000	0.300	1.000	1.000	1.001
Neutrophil-to-lymphocyte ratio	0.028	0.112	0.803	1.028	0.825	1.281

## Discussion

This single-center retrospective study analyzes the clinicopathologic features, treatment approaches, and outcomes of 52 children with osteosarcoma and Ewing sarcoma treated at a tertiary pediatric hospital in Jordan. These findings contribute to the limited body of regional research on pediatric bone sarcomas and identify key prognostic factors that may inform clinical practice and healthcare planning in resource-constrained settings. Regional data are particularly important because access to specialized sarcoma care, diagnostic delays, and resource limitations can influence stage at presentation and survival outcomes.

Ewing sarcoma accounted for 75% of cases, while osteosarcoma accounted for 25%, a distribution that differs from most international cohorts in which osteosarcoma is more common [1]. Given the single-center design and limited sample size, these proportions should be interpreted cautiously, as they may reflect referral patterns, case-mix differences, or local epidemiologic variation rather than true population-level incidence. The mean age at diagnosis was 10.5 years, and the majority of patients were male, consistent with established epidemiological patterns for pediatric bone sarcomas. This age distribution is broadly similar to that reported in prior studies, although some cohorts have shown slight variation depending on case mix and referral patterns [3].

Over one-third of patients (38.4%) presented with metastatic disease, which may reflect delayed presentation related to limited access to specialized care, misinterpretation of early symptoms, and delays in referral to tertiary centers. This proportion exceeds the 20-25% metastatic rate typically reported in large international studies of both osteosarcoma and Ewing sarcoma [6,10]. The elevated rate of metastatic presentation may reflect delays in diagnosis related to limited access to specialized care, misinterpretation of early symptoms, and referral delays from primary or secondary care settings, all of which are recognized barriers in regional pediatric oncology [11]. As metastatic disease at diagnosis was associated with a nearly ten-fold increased risk of mortality in this study, it is critical for healthcare systems in Jordan and similar contexts to prioritize reducing diagnostic delays and enhancing early recognition of bone sarcoma symptoms in both primary and secondary care settings.

The majority of primary tumors were located in the pelvis (21.2%) and lower limbs; the high frequency of pelvic tumors may have contributed to delayed diagnosis and more difficult local control, which could partly explain the poorer outcomes observed in this cohort, consistent with established patterns for bone sarcomas [12]. Pelvic tumors present particular therapeutic challenges due to the difficulty of achieving clear surgical margins, often necessitating combined surgical and radiotherapeutic approaches [13]. Surgical intervention was performed in 32/52 patients (61.5%). Among operated patients, limb-salvage surgery was the predominant procedure (29/32, 90.6%), whereas amputation was required in 3/32 (9.4%); 20/52 patients (38.5%) did not undergo surgery, likely due to unresectable tumors, extensive metastasis, or other contraindications. Recent evidence indicates that limb-salvage surgery can be as effective as amputation when complete tumor resection is feasible, although it requires a highly skilled multidisciplinary team and meticulous preoperative planning. Our finding that positive surgical margins independently predicted mortality highlights the central role of surgical margin status as a marker of local control and a major determinant of survival [14].

Relapse was observed in nine (17.3%) patients, predominantly manifesting as pulmonary metastases. The multivariable analysis did not identify significant predictors of relapse, likely due to the limited sample size. This pattern aligns with the existing literature on pediatric bone sarcomas, which reports lung metastases frequently. Rigorous surveillance, combined with surgical resection or targeted radiotherapy for isolated pulmonary relapses, may enhance outcomes for selected patients [15,16].

At the last follow-up, 39 (75%) patients in this cohort were alive, a proportion comparable to the five-year survival rates of 60-75% reported for localized pediatric osteosarcoma and Ewing sarcoma [17,18]. However, direct comparison is limited by the short and variable follow-up durations and the absence of actuarial survival estimates. The observed rates of disease progression and mortality underscore the need for collaborative clinical trials, risk-adapted therapeutic strategies, and the development of novel treatments for patients with high-risk or metastatic disease.

ALP and NLR were not significant prognostic markers in this cohort, despite previously reported associations with tumor burden, inflammation, and poor overall or disease-free survival in other pediatric sarcomas [19,20]. Larger multi-center studies are required to validate the prognostic utility of these biomarkers in regional pediatric bone sarcoma populations.

Limitations

Several limitations should be acknowledged. The retrospective, single-center design introduces potential information bias and restricts generalizability beyond the tertiary referral population. Osteosarcoma and Ewing sarcoma were analyzed collectively due to limited subgroup sizes, which precluded histology-specific comparisons. The small number of outcome events reduced statistical power for multivariable analyses. Variable and relatively short follow-up durations prevented estimation of long-term survival outcomes.

## Conclusions

Within this single-center cohort, Ewing sarcoma was the most prevalent pediatric bone sarcoma, frequently presenting with pelvic or lower limb primaries and a high metastatic burden at diagnosis. Approximately one-quarter of patients died during follow-up. Metastatic disease and positive surgical margins were identified as independent predictors of mortality.

These findings highlight the urgent need for early diagnosis, timely referral to specialized centers, and improved local control strategies. Future research should focus on multicenter trials, long-term outcome evaluation, and the development of region-specific therapeutic approaches to enhance survival in pediatric bone sarcomas.
